# Peer counselling versus standard‐of‐care on reducing high‐risk behaviours among newly diagnosed HIV‐positive men who have sex with men in Beijing, China: a randomized intervention study

**DOI:** 10.1002/jia2.25079

**Published:** 2018-02-12

**Authors:** Yu Liu, Sten H Vermund, Yuhua Ruan, Hongjie Liu, K Rivet Amico, Jane M Simoni, Bryan E Shepherd, Yiming Shao, Han‐Zhu Qian

**Affiliations:** ^1^ Department of Public Health Sciences University of Rochester School of Medicine and Dentistry Rochester NY USA; ^2^ School of Public Health Yale University New Haven CT USA; ^3^ State Key Laboratory of Infectious Disease Prevention and Control (SKLID) Collaborative Innovation Center for Diagnosis and Treatment of Infectious Diseases Chinese Center for Disease Control and Prevention Beijing China; ^4^ Department of Epidemiology and Biostatistics School of Public Health University of Maryland College Park MD USA; ^5^ Department of Health Behavior and Health Education University of Michigan Ann Arbor MI USA; ^6^ Department of Psychology University of Washington Seattle Seattle WA USA; ^7^ Department of Biostatistics Vanderbilt University School of Medicine Nashville TN USA

**Keywords:** men who have sex with men, HIV diagnosis, peer counselling, high‐risk behaviours, China

## Abstract

**Introduction:**

Reducing high‐risk behaviours (i.e. multiple partnership, condomless anal/vaginal sex, alcohol use before sex, illicit drug use) after HIV diagnosis is critical for curtailing HIV transmission. We designed an intervention to explore peer‐ counselling in reducing high‐risk behaviours among newly diagnosed HIV‐positive Chinese men who have sex with men (MSM).

**Methods:**

We randomized 367 newly diagnosed HIV‐positive men to either standard‐of‐care (SOC; n = 183) or peer‐counselling intervention (n = 184), and followed them for 12 months (visit at 0‐, 3‐, 6‐, 9‐ and 12‐month). SOC participants received counselling on high‐risk behaviour reduction by clinic staff. Intervention participants received both SOC and peer counselling. A generalized estimating equation was used to compare pre‐post diagnosis high‐risk behaviour change; logistic regression was used to assess the likelihood of practicing high‐risk behaviours between intervention and SOC participants. Both intent‐to‐treat and per‐protocol (full‐dosage) approaches were used for the analyses.

**Results:**

For pre‐ and post‐diagnosis comparisons, multiple partnership fell from 50% to 16% (*p *<* *0.001), alcohol use before sex from 23% to 9% (*p *=* *0.001), illicit drug use from 33% to 6% (*p *<* *0.001), condomless anal sex from 47% to 4% (insertive from 23% to 2%; receptive from 36% to 3%; *p *<* *0.001). In the intent‐to‐treat analysis accounting for repeated measures, peer counselling was more likely to reduce insertive anal sex (AOR = 0.65; 95% CI: 0.45 to 0.94), condomless anal sex (AOR = 0.27; 95% CI: 0.10 to 0.64) and illicit drug use (AOR = 0.32; 95% CI: 0.16 to 0.64). In the per‐protocol analysis, peer counselling was associated with a lower likelihood of using illicit drug (OR = 0.23; 95% CI: 0.07 to 0.81) and having condomless vaginal sex with women (OR = 0.12; 95% CI: 0.07 to 0.98).

**Conclusions:**

We observed a 14 to 43% decrease in the prevalence of selected high‐risk behaviours after HIV diagnosis. Peer counselling had a greater impact in reducing condomless anal sex with men, illicit drug use and condomless vaginal sex with women over time. Future studies with exclusive peer‐counselling arm are necessary to test its efficacy and effectiveness among Chinese MSM.

Clinical Trial Number: NCT01904877

## 1 Introduction

In China, an inexorable upward trend of HIV has been witnessed among men who have sex with men (MSM) 1, 2, 3, with the HIV prevalence among this subgroup surging from 1.2% in 2005 to 7.7% in 2014 4. A mathematical modelling also suggests a potential escalation of HIV prevalence among Chinese to 21.4% by 2020, if no further effective prevention interventions are implemented. 5. The unrelenting HIV burden among Chinese MSM highlights an urgent need to design and implement innovative, feasible, and effective HIV interventions.

Timely HIV diagnosis and rapid post‐diagnosis linkage‐to‐care is a critical step of the continuum of HIV care 6. The theory behind this testing‐and‐linkage‐to‐care strategy involves “prevention with positives,” including antiretroviral therapy (ART) initiation/adherence for viral suppression and safer sex through behavioural interventions to reduce transmission to others 7, 8, 9. Despite the scale‐up in available and free HIV care services in recent years, Chinese MSM remain suboptimal in regular HIV testing, linkage to and engagement in HIV care 10, 11. On one hand, this challenge can be largely attributed to various individual and structural barriers reported among Chinese MSM, including low HIV literacy, HIV‐associated stigma, cultural discrimination against homosexuality, lack of support, HIV disclosure concern and psychological burden. On the other hand, the authoritativeness of regular healthcare provider and the rigidness of universal China Center for Disease Control and Prevention (CDC) standard‐of‐care (hereafter SOC) may be insufficient to accommodate specific HIV care needs of Chinese MSM, resulting in reduced confidence and trust in routine HIV care 10, 11, 12, 13.

Peers are often effective changing agents among social/sexual minority subgroups 14. As supporting members and role models to their community, peers are also capable of outreaching to specific hidden populations and facilitating care delivery to those in need 15. Compared to regular healthcare professionals, peer educators/counsellors may be more cost‐effective 16 in influencing behavioural modifications by addressing clients’ psychological needs without discrimination or stigmatization 16. Qualitative evidence suggests that peer counselling may help to build confidence and trust towards HIV care services 11, 17. A meta‐analysis of fifteen studies also found peer‐led HIV interventions might reduce overall unprotected anal intercourse among HIV‐negative MSM. However, most interventions were conducted in American and Canada (n = 13), were not randomized control trials (RCT) (n = 13) and were simply group‐based (n = 15) 18. Very little is known regarding the efficacy of individual‐based peer counselling intervention in reducing high‐risk among HIV‐positive MSM.

The promising peer‐delivered counselling for safer sex can be a crucial and scalable alternative to the predominant SOC to foster better HIV care engagement among Chinese MSM. However, no RCT has ever been conducted to compare the efficacy of peer counselling verses SOC in reducing high‐risk behaviours among HIV‐positive Chinese MSM. We conducted the first one‐on‐one randomized intervention trial in Beijing, China to (1) to assess change in high‐risk behaviours across a cohort of recently diagnosed HIV‐positive Chinese MSM before and after diagnosis; and (2) in the same cohort, assess difference in high‐risk behaviours between those randomized to a 4‐session peer counselling intervention vs. those randomized to SOC counselling during a 12‐month follow‐up.

## 2 Methods

### 2.1 Study design and setting

This study was a 2‐year (2013.3 to 2015.3), two‐phase RCT assessing the efficacy of peer‐counselling vs. SOC on ART initiation/adherence (primary outcome of the trial) as well as high‐risk behaviours change (main outcome in the current study), quality of life, HIV stigma, self‐efficacy, hospital anxiety and depression among newly diagnosed HIV‐positive MSM in Beijing, China 19, 20. Phase I was a continuing enrolment phase that has been substantially described elsewhere 10, 21, 22, 23, 24, 25. In short, we collaborated with a local gay‐friendly community‐based organization (CBO; Chaoyang AIDS Volunteer Group) to recruit participants via short message service, website advertisement, gay‐frequented venue outreach and peer referral. The inclusion criteria include: cis‐gender man, had sex with another man in the past 12 months, 18 years or older, living in Beijing and not planning to relocate in the next 12 months, HIV‐negative or status‐unknown (self‐report), willing to provide blood sample for HIV test, and willing to provide written informed consent for study participation. Eligible participants were asked to complete a questionnaire survey on socio‐demographic characteristics and behavioural risk factors, followed by a free onsite HIV rapid test. Participants with an initial positive result were asked to have their blood drawn for laboratory confirmatory tests. Participants were invited back to the clinic within 5 days of the initial visit receive lab test result informed by a medical doctor.

HIV‐positive men consented to participate in Phase II trial were randomized to receive either peer counselling or SOC within 1 week of their HIV diagnosis delivery. Treatment assignments generated by Vanderbilt Data Coordinating Center were put in numbered envelopes to be opened by study participants upon randomization. To ensure a balanced randomization between intervention and SOC arm, participants were first block‐randomized using a 1:1 ratio to each of the two collaborated HIV clinics (Xicheng District CDC or Chaoyang District CDC). In each clinic, an equal number of participants were further randomized to intervention or SOC arm in multiple 4‐ or 6‐person block sizes until the balance was reached 26. Randomized participants were directed to visit the designated CDC clinic to complete a survey and the first counselling to begin the Phase II trial. Participants in both arms were followed for 12 months, and were requested to complete either peer or SOC counselling at 0‐, 3‐, 6‐ and 9‐month with a follow‐up survey at 3‐, 6‐, 9‐ and 12‐month. The questionnaire survey was set to precede the counselling during a visit with both events (0‐, 3‐, 6‐ and 9‐month). We allowed a “buffering period” (±7 days before/after the scheduled date) to facilitate the participant retention.

SOC participants received counselling provided by a CDC‐trained doctor. A generic message reminder was sent to the participants 3 days prior to the scheduled visit. The 30‐min SOC counselling covered contents related to safer sex and prevention of HIV transmission per China CDC's HIV counselling guidelines. SOC counsellor also addressed questions/concerns from the participants during the visit. In peer counselling, a total of six peer counsellors were recruited and trained before administering peer counselling. All peer counsellors were HIV‐positive MSM who had at least 6 months of experience in community‐based HIV counselling services, received 6‐hour manual‐guided workshop training, and 4‐hour individual face‐to‐face training from an experienced behavioural scientist (K.R.A.). The peer‐counselling manual was developed and enhanced based on an adapted Information‐Motivation‐Behavioural Skills (IMB) model 27, 28, 29, 30, 31. Participants in the peer‐counselling intervention arm would receive a message sent by the designated peer counsellor via cellphone or social media apps to schedule a mutually confirmed time/date within the buffering period. The peer‐counselling session involved a one‐on‐one 60‐minute discussion focusing on topics regarding specific high‐risk behaviours modification, including the strategy to reduce male/female sexual partners, condomless anal/oral sex, commercial sex, illicit drug use, alcohol intoxication and multiple concurrent partnerships. At the end of the counselling, peer counsellor and participant would identify one or more goals for safer sex to be qualitatively evaluated in the next visit. As mandated by China CDC, all participants in the peer‐counselling intervention arms need to attend routine SOC. Thus, participants in the intervention arm received an initial SOC prior to the peer‐counselling session. The study protocol was reviewed and approved by the institutional review boards of Vanderbilt University, and the National Center for AIDS/STD Control and Prevention (NCAIDS) of China Center for Disease Control and Prevention.

### 2.2 Data collection

A trained nurse in the clinic administered a paper‐based questionnaire survey to all participants upon enrolment (before HIV testing) to collect data on: 1 socio‐demographic characteristics, including age, ethnicity, marital status, education, employment, income, legal household registration in Beijing (*hukou*), duration of living in Beijing, and health insurance status; 2 pre‐diagnosis high‐risk behaviours, including age of sex debut, year of sexual activity, lifetime male/female sexual partners, prior HIV testing histories, recent (past‐3‐month) alcohol consumption, recent alcohol use before sex, recent illicit drug use (self‐reported intake of any of these drugs: methamphetamine, MDMA, rush, magu [a stimulant consisting of Methamphetamine and caffeine], ketamine, cannabis/marijuana, cocaine, opium, heroin or morphine), recent condomless insertive/receptive anal sex with men, recent sex with women, recent experience with male commercial sex workers, recent anal or oral sex with HIV‐positive men, and self‐reported HIV risk perception 22, 24. HIV‐positive participants who continued Phase II trial were asked to further complete a nurse‐administered questionnaire on past‐3‐month high‐risk behaviours (ascertained at 3‐, 6‐, 9‐ and 12‐month follow‐ups) and other interested outcomes (ascertained at 0‐, 3‐, 6‐, 9‐ and 12‐month follow‐ups), including quality of life 32, stigma to HIV 33 and homosexuality 34, self‐efficacy 35, hospital anxiety and depression 36, 37. All data regarding linkage‐to‐care and ART initiation analysed elsewhere were obtained from medical records and National ART Database 19, 20. Both HIV and syphilis tests were lab‐confirmed. All laboratory tests and techniques have been described elsewhere 24, 38.

### 2.3 Statistical analyses

To compare the baseline characteristics between the intervention and SOC arms, we used Chi‐square or Fisher's exact tests for categorical variables and Wilcoxon Rank‐sum tests for continuous variables. We used generalized estimating equations (GEE) to assess the statistical significance of high‐risk behaviour change pre‐ and post‐HIV diagnosis, and to assess the trends of these behavioural changes during the 12 months follow‐up, adjusted for a priori confounders (age, ethnicity, education, marital status and employment). ART use is suggested to be associated with high‐risk behaviour change and healthcare visits 39. In our study, more than half of the participants initiated ART during the first 3 months of follow‐up. Therefore, we also adjusted ART use as a confounder in the current analysis. For intent‐to‐treat analysis, we performed bivariate and multivariable GEE models with logit function 40 to longitudinally assess the impact of peer counselling vs. SOC in selected significant high‐risk behaviours (*p *<* *0.05 in Table S1). In the multivariable GEE model, we adjusted for corresponding baseline high‐risk behaviour, age, ethnicity, education, marital status, employment and ART use. In the per‐protocol analysis (full‐dosage analysis), we conducted subgroup analyses among participants in both arms that completed all four sessions of either SOC or peer counselling. Unconditional logistic regression analyses were employed to compare selected high‐risk behaviours among participants between intervention and SOC arm based on the 12‐month exit survey data. Due to insufficient degrees of freedom to spend (sparse “yes” response in the characteristics being evaluated), only unadjusted result is presented to avoid over‐fitting when performing multivariable adjustment 41. We used Stata 12.0™ (StataCorp LP, College Station, Texas, USA) for all statistical analyses.

## 3 Results

### 3.1 Enrollment and study population

**Figure 1:**
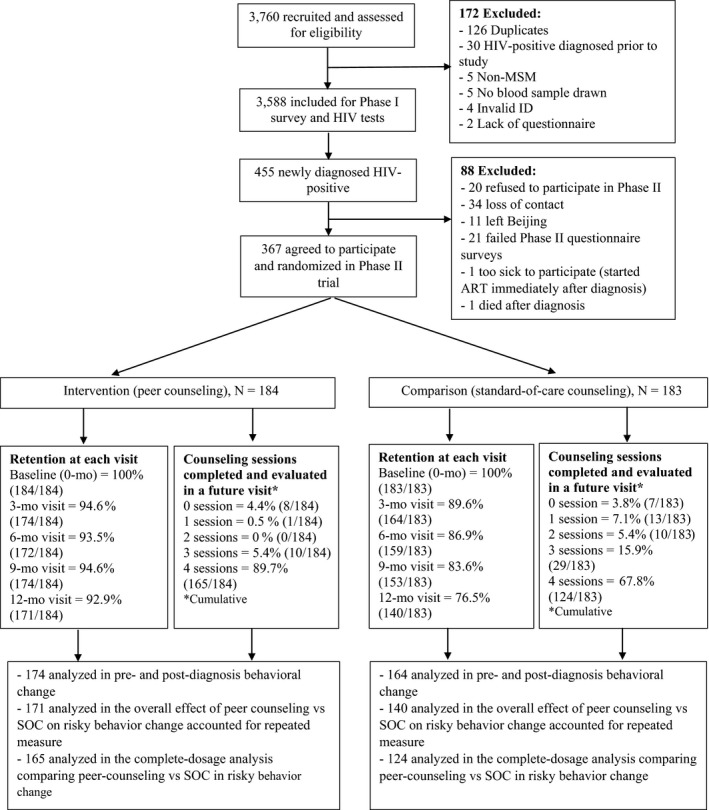
Screening, enrollment flow, retention and intervention dosage among study participants in the trial.

We initially recruited 3760 MSM in Phase I and then excluded 172 observations due to duplicate participation, confirmed HIV‐seropositivity prior to this study, non‐MSM, refusal to provide blood sample, invalid identification number, or lack of a questionnaire. Therefore, 3588 participants undertook HIV tests and 455 men were newly diagnosed as being HIV‐positive (prevalence = 12.7%; 455/3588). Among the 455 HIV‐positive MSM, 367 (367/455; 80.7%) were further enrolled to Phase II randomization to either intervention (n = 184) or control (n = 183) arms. Eighty‐eight participants were excluded due to refusal to participate in Phase II, loss of contact, having left Beijing for hometown for treatment after diagnosis, failure to fulfil Phase II questionnaires, very poor health, or death after diagnosis. Details of the enrolment and retention process are illustrated in Figure 1.

Socio‐demographic characteristics of the 3588 participants in Phase I have been described elsewhere 21, 24.Among the 367 HIV‐positive men entering the Phase II trial, the median age was 28 years (interquartile range, 24 to 32). Most men were Han‐ethnic (93%), single (88%), employed (83%), non‐local residents (82%), and college‐educated (77%). The post‐randomization distribution of most baseline characteristics were not significantly (*p *>* *0.05) different between two arms (Table 1).

**Table 1:** Baseline characteristics of newly diagnosed HIV‐positive Chinese MSM in a randomized clinical trial (N = 367)

Characteristics	ALL (N = 367) n, (%)	Intervention arm (N = 184) n, (%)	Standard‐of‐care arm (N = 183) n, (%)	*P*‐value
Age, year				0.91
Median, IQR	28, (24–32)	28, (25–32)	28, (24‐33)	
Ethnicity				0.31
Han majority	342 (93.2)	169 (91.8)	173 (94.5)	
Non‐Han minorities	25 (6.8)	15 (8.2)	10 (5.5)	
Current marital status				0.10
Unmarried	323 (88.0)	167 (90.8)	156 (85.2)	
Married	44 (12.0)	17 (9.2)	27 (14.8)	
Education (year of schooling)				0.26
College and above (>12)	282 (76.8)	149 (81.0)	133 (72.7)	
Senior high school 10, 11, 12	35 (9.6)	15 (8.2)	20 (10.9)	
Junior middle school 7, 8, 9	46 (12.5)	19 (10.3)	27 (14.8)	
Primary school or lower (≤6)	4 (1.1)	1 (0.5)	3 (1.6)	
Employment				0.25
Employed	304 (82.8)	157 (85.3)	147 (80.3)	
Unemployed/retired	25 (6.8)	10 (5.4)	15 (8.2)	
Student	24 (6.6)	13 (7.1)	11 (6.0)	
Other	14 (3.8)	4 (2.2)	10 (505)	
Monthly income, Chinese Yuan (1 USD ≈ 6.8 Yuan)				0.76
Median, IQR	5000 (3000 to 8000)	5000 (3000 to 8000)	5000 (3000 to 8000)	
Health insurance				0.08
No	164 (44.7)	74 (40.2)	90 (49.2)	
Yes	203 (33.5)	110 (59.8)	93 (50.8)	
Legal Beijing residency (*Hukou*)				0.81
No	301 (82.0)	150 (81.5)	151 (82.5)	
Yes	66 (18.0)	34 (18.5)	32 (17.5)	
Duration of living in Beijing, year				0.97
Median, IQR	5, (2 to 10)	5, (2 to 9)	5, (2 to 10)	
Age of sex debut, year				0.76
Median, IQR	20, (18 to 22)	20, (18 to 22)	20, (18 to 23)	
Year of sexual activity				0.88
Median, IQR	8, (4 to 12)	8, (4 to 11)	8, (4 to 12)	
Lifetime number of male sexual partners				0.16
<10	159 (43.3)	73 (39.7)	86 (47.0)	
≥10	208 (56.7)	111 (63.3)	97 (53.0)	
Perception of HIV risk prior to HIV diagnosis				0.06
Low or very low	100 (27.3)	42 (22.8)	58 (31.7)	
High or very high	267 (72.7)	142 (77.2)	125 (68.3)	
Syphilis infection				0.37
No	313 (85.3)	160 (87.0)	153 (83.6)	
Yes	54 (14.7)	24 (13.0)	30 (16.4)	

### 3.2 Pre‐ and post‐ HIV diagnosis high‐risk behaviours change

Table 2 shows the prevalence of recent high‐risk behaviours before and after HIV diagnosis. Comparing to baseline survey, reported risk behaviours assessed at 3‐month declined dramatically, including alcohol consumption (55.1% vs. 39.1%; *p *<* *0.001), alcohol use before sex (22.9% vs. 8.6%; *p *<* *0.001), illicit drug use (33.0% vs. 5.9%; *p *<* *0.001), having had multiple male sexual partners (50.1% vs. 15.7%; *p *<* *0.001), having had condomless anal sex (47.4% vs. 3.8%; *p *<* *0.001) with men, having had condomless insertive anal sex (23.4% vs. 2.1%; *p *<* *0.001) with men, and having had condomless receptive anal sex (36.2% vs. 3.0%; *p *<* *0.001) with men. The decreasing pattern for these behaviours was consistent after stratifying in study arms.

**Table 2:** Comparison of high‐risk behaviours during the past 3 months assessed before and after HIV diagnosis among Chinese MSM in a randomized clinical trial

	Overall			Intervention arm			Standard‐of‐care arm		
Characteristics	Pre HIV diagnosis (N = 367) n (%)	Post HIV diagnosis (N = 338) n (%)	*p*‐value	Pre HIV diagnosis (N = 184) n (%)	Post HIV diagnosis (N = 174) n (%)	*p*‐value	Pre HIV diagnosis (N = 183) n (%)	Post HIV diagnosis (N = 164) n (%)	*p*‐value
Alcohol consumption			<0.001			<0.001			<0.001
No	165 (44.9)	206 (60.9)		82 (44.6)	111 (63.8)		83 (45.4)	95 (57.9)	
Yes	202 (55.1)	132 (39.1)		102 (55.4)	73 (36.2)		100 (54.6)	69 (42.1)	
Alcohol use before sex			<0.001			0.003			0.001
No	283 (77.1)	309 (91.4)		147 (79.9)	159 (91.4)		136 (74.3)	150 (91.5)	
Yes	84 (22.9)	29 (8.6)		37 (20.1)	15 (8.6)		47 (25.7)	14 (8.5)	
Illicit drug use			<0.001			<0.001			<0.001
No	246 (67.0)	318 (94.1)		132 (71.6)	168 (96.9)		114 (62.5)	150 (91.4)	
Yes	121 (33.0)	20 (5.9)		52 (28.4)	6 (3.1)		69 (37.5)	14 (8.6)	
Had multiple (>1) male sexual partners			<0.001			<0.001			<0.001
No	183 (49.9)	285 (84.3)		95 (51.6)	148 (85.1)		88 (48.1)	137 (83.5)	
Yes	184 (50.1)	53 (15.7)		89 (48.4)	26 (14.9)		95 (51.9)	27 (16.5)	
Had condomless anal sex with men			<0.001			<0.001			<0.001
No	193 (52.6)	325 (96.2)		96 (52.2)	171 (98.3)		97 (53.0)	154 (93.9)	
Yes	174 (47.4)	13 (3.8)		88 (47.8)	3 (1.7)		86 (47.0)	10 (6.1)	
Had condomless insertive anal sex with men			<0.001			<0.001			<0.001
No	281 (76.6)	331 (97.9)		139 (75.5)	171 (98.3)		142 (77.6)	160 (97.6)	
Yes	86 (23.4)	7 (2.1)		45 (24.5)	3 (1.7)		41 (22.4)	4 (2.4)	
Had condomless receptive anal sex with men			<0.001			<0.001			<0.001
No	234 (63.8)	328 (97.0)		114 (62.0)	173 (99.4)		120 (65.6)	155 (94.5)	
Yes	133 (36.2)	10 (3.0)		70 (38.0)	1 (0.6)		63 (34.4)	9 (5.5)	
Had anal sex with known HIV‐positive men			0.15			0.57			0.14
No	351 (95.6)	315 (93.2)		175 (95.1)	163 (93.7)		176 (96.2)	152 (92.7)	
Yes	16 (4.4)	23 (6.8)		9 (4.9)	11 (6.3)		7 (3.8)	12 (7.3)	
Had oral sex with known HIV‐positive men			0.14			0.08			0.87
No	351 (95.6)	330 (97.6)		174 (94.6)	171 (98.3)		177 (96.7)	159 (97.0)	
Yes	16 (4.4)	8 (2.4)		10 (5.4)	3 (1.7)		6 (3.3)	5 (3.0)	
Had anal sex with commercial sex worker			0.66			0.37			0.88
No	360 (98.1)	333 (98.5)		181 (98.4)	173 (99.4)		179 (97.8)	160 (97.6)	
Yes	7 (1.9)	5 (1.5)		3 (1.6)	1 (0.6)		4 (2.2)	4 (2.4)	
Had condomless vaginal sex with women			0.02			0.04			0.17
No	350 (95.4)	331 (97.9)		176 (95.6)	171 (98.3)		174 (95.1)	160 (97.6)	
Yes	17 (4.6)	7 (2.1)		8 (4.4)	3 (1.7)		9 (4.9)	4 (2.4)	

Sample size may vary due to missing response and attrition from baseline to 3‐month follow‐up visit.

### 3.3 Trend and overall impact of peer counselling vs. SOC

**Figure 2:**
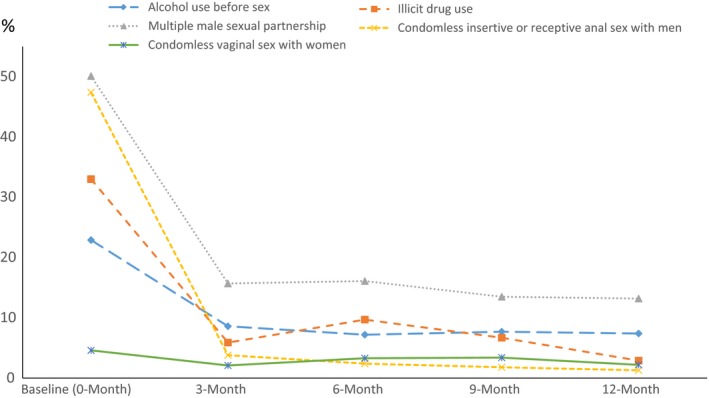
Trends of selected high‐risk behaviors across baseline (pre‐diagnosis, 0‐month) and post‐diagnosis (3‐, 6‐, 9‐ and 12‐month) visits among newly‐diagnosed HIV‐positive Chinese MSM in Beijing, China.

Figure 2 displays the trend of selected high‐risk behaviours over the study timeframe. In contrast to the sharp decrease immediately post‐diagnosis (Table 2), we saw a plateau in the prevalence of almost all sexual and drug‐using behaviours starting from the 3‐month to the end of the study. Neither peer‐counselling intervention nor SOC alone further reduced the prevalence of these high‐risk behaviours beyond that was seen immediately post‐diagnosis (*p*_trend _> 0.05). Peer counselling was more likely to reduce the frequency of practicing insertive anal sex, condomless receptive or insertive anal sex and illicit drug use over the 12 months period (*p *<* *0.05) (Table S1). In multivariable analysis using intent‐to‐treat approach (Table 3), compared to SOC, MSM receiving peer counselling intervention had a 35% reduced risk of were practicing insertive anal sex with men (AOR: 0.65; 95% CI: 0.44 to 0.91), 73% reduced risk of engaging in condomless anal sex with men (AOR: 0.27; 95% CI: 0.10 to 0.64), and 68% reduced risk of using illicit drugs (AOR: 0.32; 95% CI: 0.16 to 0.64).

**Table 3:** Bivariate and multivariable logistic regression analyses of the impact of peer counselling intervention versus standard‐of‐care on selected sexual behaviours among Chinese MSM over a 12‐month follow‐up (intention‐to‐treat)

High risk behaviors	Comparison	OR	95% CI	aOR^a^	95% CI
Had insertive anal sex with men	Yes vs. no	0.63	(0.44, 0.91)	0.65	(0.45, 0.94)
Had condomless receptive or insertive anal sex with men	Yes vs. no	0.25	(0.09, 0.65)	0.27	(0.10, 0.74)
Illicit drug use	Yes vs. no	0.29	(0.15, 0.59)	0.32	(0.16, 0.64)

OR, odds ratio; aOR, adjusted odds ratio; CI, confidence interval.

a Adjusted for the corresponding baseline high‐risk behaviour, age, ethnicity, education, marital status, employment and ART use.

### 3.4 Subgroup analysis of full‐dosage receiving participants

Results of the logistic regression analyses of associations between counselling status (intervention vs. SOC) and high‐risk behaviours among full‐dosage (completion of all four counselling sessions in either arm) participants are shown in Table 4. Based on the 12‐month exit survey, we found participants in the peer counselling arm had a 77% lower risk of using illicit drug (OR = 0.23; 95% CI: 0.07 to 0.81) and 88% lower risk of practicing condomless vaginal sex with women (OR = 0.12; 95% CI: 0.02 to 0.98) compared to those receiving SOC counselling alone.

**Table 4:** Subgroup analysis of peer counselling versus standard‐of‐care on recent high‐risk behaviours among newly diagnosed HIV‐positive MSM participated in all sessions (per‐protocol^a^)

	Intervention (N = 165)	Standard‐of‐care (N = 124)	
Characteristics	n (%)	n (%)	Unadjusted OR (95% CI)*
Alcohol consumption			
No	102 (61.8)	76 (61.3)	Reference
Yes	63 (38.2)	48 (38.7)	0.97 (0.61, 1.58)
Alcohol use before sex			
No	151 (91.5)	116 (93.6)	Reference
Yes	14 (8.5)	8 (6.4)	1.34 (0.55, 3.31)
Illicit drug use			
No	149 (97.6)	121 (90.3)	Reference
Yes	16 (2.4)	3 (9.7)	**0.23 (0.07, 0.81)**
Had multiple (>1) male sexual partners			
No	146 (88.5)	103 (83.1)	Reference
Yes	19 (11.5)	21 (16.9)	0.64 (0.33, 1.25)
Had condomless insertive or receptive anal sex with men			
No	163 (98.8)	122 (98.4)	Reference
Yes	2 (1.2)	2 (1.6)	0.75 (0.11, 5.39)
Had anal sex with known HIV‐positive men			
No	155 (96.9)	120 (96.8)	Reference
Yes	10 (6.1)	4 (3.2)	1.94 (0.59, 6.32)
Had oral sex with known HIV‐positive men			
No	160 (97.0)	123 (99.2)	Reference
Yes	5 (3.0)	1 (0.8)	3.84 (0.44, 33.32)
Had anal sex with commercial sex worker			
No	163 (98.8)	124 (100)	Reference
Yes	2 (1.2)	0	–
Had condomless vaginal sex with women			
No	164 (99.4)	118 (95.2)	Reference
Yes	1 (0.6)	6 (4.8)	**0.12 (0.02, 0.98)**

OR, odds ratio; CI, confidence interval.

a Assessed cross‐sectionally using the last follow‐up data (12‐month visit).

## 4 Discussion

In our Beijing MSM study, we observed a 14 to 43% decreased prevalence in alcohol drinking before sex, illicit drug use, multiple male partnerships and condomless anal sex among HIV‐positive Chinese MSM after an HIV diagnosis. In contrast, anal/oral sex with HIV‐positive MSM, commercial sex, and condomless sex with women was less frequent in this population, thus not varying at a greater extent in pre‐ and post‐ HIV diagnosis comparison (0.4 to 2.4% decrease). Our findings were consistent with a longitudinal study among MSM in Amsterdam showing that recently seroconverted MSM reduce their condomless anal sex and number of sexual partners following an HIV diagnosis 42. A Mathematical modelling study also reveals substantial risky behaviour reduction among post‐diagnosis MSM in Southern California 43. However, a recent study in Los Angeles indicates a higher prevalence of condomless anal sex among recently seroconverted MSM compared to that before HIV diagnoses 44. Per the China CDC guidelines, our study participants received both pre‐test and post‐test counselling in safer sex provided by healthcare staff; participants in the intervention arm received both SOC and peer counselling. This may explain the substantial decline in risky behaviours that participants reported post‐diagnosis. Alternatively, awareness of one's HIV positivity status may raise psychological distress and reduce motivation for sex 45. It is challenging to disaggregate whether high‐risk behaviours were reduced among HIV‐positive MSM as a result of the HIV diagnosis itself or due to the impact of pre‐ and post‐test counselling; or the dramatic decrease was simply a phenomenon of “regression to the mean” 46 in a prospective intervention study. Future trial with minimum yet ethical pre‐ and post‐test safer sex counselling may help better elucidate the influence of HIV diagnosis on modifying high‐risk behaviours.

Previous studies among HIV‐negative Chinese MSM by Zhang et al. (serial cross‐sectional study) and Zhu et al.(single arm pre‐post intervention) suggests that intervention based on MSM peer groups is feasible to reduce unprotected anal intercourse 47, 48. In our study among newly diagnosed HIV‐positive Chinese MSM in Beijing, we found peer counselling (vs. clinic staff‐delivered SOC) intervention were more likely to reduce illicit drug use, condomless anal sex with men and condomless vaginal sex with women in the 12 months post‐diagnosis. Our per protocol analyses (vs. intent‐to‐treat) showed a greater peer‐counselling intervention effect on risky behaviour (i.e. illicit drug use) reduction compared to SOC, suggesting a cumulative benefit of peer counselling in positively influencing risky behaviours. In line with our findings, an RCT among HIV‐infected MSM in Chicago, United States found greater HIV transmission risk reduction in an treatment advocacy programme led by peer advocates compared to those in standard care 49. Aggregate evidence of 22 MSM studies suggests that peer‐delivered intervention is more efficacious in discouraging high‐risk activities or encouraging safer sex practice among MSM 18. Compared to traditional healthcare providers, peers are more credible and less stigmatizing or judgmental, which is essential in building mutual trust before any counselling can be successfully delivered 14, 18. An experimental analysis among an urban gay community centre also suggests that peer educators/counsellors are often deemed as opinion leader among gay community members; interventions that are employed and endorsed by such roles may less high‐risk takings 50.

Although peer counselling was found to be effective in reducing several high‐risk behaviours in our trial, the overall results should be interpreted critically. Nearly all high‐risk behaviours fell dramatically between baseline and 3‐month visit, but did not show any greater reducing trend over time in both arms. This might be due to the strengthened SOC guided by the “Four Free, One Care” policy 17, thus the SOC counselling corresponded with our trial time period might have been effective in nurturing risk reduction, especially relevant to our participating clinics in Beijing where SOC staff received additional centralized training before study participation. It is also likely that since both intervention and control arms received SOC counselling, any additional efficacy as a result of peer counselling intervention might be too small to be measured apart from the existing SOC; or the robust SOC effect is likely to compensate the effect of any peer counselling. Finally, the counselling may not be a principal factor in the reduction in high‐risk activities post‐diagnosis. Prevention services offered to HIV‐positive peers are a recognized strategy to reduce new infections 51, along with suppression of viral load by early ART initiation and adherence 52, 53. Innovative and culturally adapted prevention interventions for “prevention with positives” remain limited among Chinese MSM, highlighting the urgent need of developing such programmes for tackling the HIV epidemics among Chinese MSM in any non‐interventional settings.

Our study is subjected to limitations. First, despite we assigned participants to a specific peer counsellor at the beginning of the trial, participants might switch to an alternative peer counsellor when the assigned peer counsellor was absent due to any emergency, which might affect peer‐counselling consistency. In addition, since the actual peer‐counselling experience was one‐on‐one between counsellor and participants, we could not monitor the actual intervention quality beyond the initial protocol‐based training (lack of fidelity monitor). Second, since the sample size was derived to test the intervention on primary outcomes (i.e. ART initiation/adherence); we were restrained in statistical power to perform a number of dosage‐specific analyses or complex multivariable adjustment in regression analyses. Third, per the trial protocol, we were limited in testing the exclusive effect of peer counselling in modifying high‐risk behaviour without the interference by SOC or introduction of ART. Fourth, high‐risk sexual and substance use behaviours are sensitive topics to our study participants. We did not use computer‐assisted self‐interviews due to the restrained resource in our study locations, subjecting our data to potential social disability bias. We also observed higher retention rates among participants in the intervention arm; the differential retention and lost‐to‐follow‐up across groups is likely to result in biased findings. However, we did not find any statistically significant difference in baseline socio‐demographic and behavioural characteristics between those retained and lost‐to‐follow‐up (data now shown). Last, this study was conducted among MSM sampled in Beijing, China, which may limit the generalizability of our findings to MSM in other regions of the country. Despite these limitations, our study was the first RCT to prospectively assess the impact HIV‐diagnosis, as well as peer counselling versus standard‐of‐care on high‐risk behaviour changes among newly diagnosed HIV‐positive Chinese MSM.

## 5 Conclusions

Much has been written about peer counselling and “positive prevention” for HIV‐infected persons. We think it is important to address the oversimplified issue of the benefits of peer counselling in various settings and among populations of different characteristics. The use of peers as facilitators for MSM‐specific intervention should be studied further, given their potential to accommodate the psychological, social, and institutional needs by MSM. Future randomized clinical trial with exclusive peer counselling arm vs. routine care or a combination of other comparable risk reduction programmes are necessary to further demonstrate its efficacy in real‐word practice.

## Competing interests

The authors report no competing interests.

## Authors’ contributions

SHV, YR, HL, KRA, JMS, BES, YS and HZQ designed the study. YL and HZQ chose the main directions for data analysis, and YL performed the statistical analysis. YL, SHV and HZQ wrote the manuscript with input from YR, HL, KRA, JMS, BES, YS. All authors revised and approved the manuscript before submission.

## Funding Information

This work was supported by grants from the National Institute of Allergy and Infectious Diseases, National Institutes of Health (R01AI094562, R34AI091446), and Tennessee Center for AIDS Research (P30 AI110527).

## Supporting information


**Table S1.** Trends for high‐risk behaviours at each post‐diagnosis follow‐up visit in a clinical trial of men who have sex with men in China.
