# Supplementary material for: Peer counselling versus standard‐of‐care on reducing high‐risk behaviours among newly diagnosed HIV‐positive men who have sex with men in Beijing, China: a randomized intervention study
Source: J Int AIDS Soc. 2018 Feb 12;21(2):e25079. doi: 10.1002/jia2.25079 (PMC5808102; doi:10.1002/jia2.25079)
Supplement: Supplementary file 1 — Table S1. Trends for high‐risk behaviours at each post‐diagnosis follow‐up visit in a clinical trial of men who have sex with men in China. [file JIA2-21-e25079-s001.docx]

**Supplemental Table 1** Trends for high-risk behaviors at each post-diagnosis follow-up visit in a clinical trial of men who have sex with men in China

| Risk factors | 3-month, n (%) | | | 6-month, n (%) | | | 9-month, n (%) | | | 12-month, n (%) | | | *P*-value ^a^ | *P*-value ^b^ | *P*-value ^c^ | *P*-value ^d^ |
| --- | --- | --- | --- | --- | --- | --- | --- | --- | --- | --- | --- | --- | --- | --- | --- | --- |
|  | Overall  (N=338) | Intervention (N=174) | SOC (N=164) | Overall  (N=331) | Intervention (N=172) | SOC (N=159) | Overall  (N=327) | Intervention (N=174) | SOC (N=153) | Overall  (N=311) | Intervention (N=171) | SOC (N=140) |  |  |  |  |
| **Alcohol consumption** |  |  |  |  |  |  |  |  |  |  |  |  | 0.96 | 0.93 | 0.74 | 0.19 |
| Never | 206 (61.0) | 111 (63.8) | 95 (57.9) | 195 (58.9) | 106 (61.6) | 89 (56.0) | 196 (59.9) | 111 (63.8) | 85 (55.6) | 189 (60.8) | 106 (62.0) | 83 (59.3) |  |  |  |  |
| Ever | 132 (39.0) | 63 (36.2) | 69 (42.1) | 136 (41.1) | 66 (38.4) | 70 (44.0) | 131 (40.1) | 63 (36.2) | 68 (44.4) | 122 (39.2) | 65 (38.0) | 57 (40.7) |  |  |  |  |
| **Alcohol use before sex** |  |  |  |  |  |  |  |  |  |  |  |  | 0.88 | 0.98 | 0.69 | 0.31 |
| No | 309 (91.4) | 159 (91.4) | 150 (91.5) | 307 (92.8) | 157 (91.3) | 150 (94.3) | 302 (92.3) | 159 (91.4) | 143 (93.5) | 288 (92.6) | 156 (91.2) | 132 (94.3) |  |  |  |  |
| Yes | 29 (8.6) | 15 (8.6) | 14 (8.5) | 24 (7.2) | 15 (8.7) | 9 (5.7) | 25 (7.7) | 15 (8.6) | 10 (6.5) | 23 (7.4) | 15 (8.8) | 8 (5.7) |  |  |  |  |
| **Illicit drug use** |  |  |  |  |  |  |  |  |  |  |  |  | 0.09 | 0.29 | 0.37 | 0.001 |
| No | 318 (94.1) | 168 (96.9) | 150 (91.4) | 299 (90.3) | 162 (94.3) | 137 (86.6) | 305 (93.3) | 168 (97.4) | 137 (89.7) | 290 (93.3) | 166 (97.1) | 124 (90.1) |  |  |  |  |
| Yes | 20 (5.9) | 6 (3.1) | 14 (8.6) | 32 (9.7) | 10 (5.7) | 22 (13.4) | 22 (6.7) | 6 (2.6) | 16 (10.3) | 21 (6.7) | 5 (2.9) | 16 (9.9) |  |  |  |  |
| **Had multiple (>1) male sexual partners** |  |  |  |  |  |  |  |  |  |  |  |  | 0.25 | 0.26 | 0.64 | 0.71 |
| No | 285 (84.3) | 148 (85.1) | 148 (85.1) | 278 (84.0) | 144 (83.7) | 134 (84.3) | 283 (86.5) | 149 (85.6) | 134 (87.6) | 270 (86.8) | 152 (88.9) | 118 (84.3) |  |  |  |  |
| Yes | 53 (15.7) | 26 (14.9) | 26 (14.9) | 53 (16.1) | 28 (16.3) | 25 (15.7) | 44 (13.5) | 25 (14.4) | 19 (12.4) | 41 (13.2) | 19 (11.1) | 22 (15.7) |  |  |  |  |
| **Had anal sex with men** |  |  |  |  |  |  |  |  |  |  |  |  | 0.48 | 0.84 | 0.25 | 0.20 |
| No | 212 (62.7) | 118 (67.8) | 94 (57.3) | 210 (63.4) | 114 (66.3) | 96 (60.4) | 214 (65.4) | 114 (65.5) | 100 (65.4) | 210 (67.5) | 117 (68.4) | 93 (66.4) |  |  |  |  |
| Yes | 126 (37.3) | 56 (32.2) | 70 (42.7) | 121 (36.6) | 58 (33.7) | 63 (39.6) | 113 (34.6) | 60 (34.5) | 53 (34.6) | 101 (32.5) | 54 (31.6) | 47 (33.6) |  |  |  |  |
| **Had insertive anal sex with men** |  |  |  |  |  |  |  |  |  |  |  |  | 0.30 | 0.96 | 0.03 | 0.01 |
| No | 256 (75.7) | 144 (82.8) | 112 (68.3) | 260 (78.6) | 143 (83.1) | 117 (73.6) | 262 (80.1) | 145 (83.3) | 117 (76.5) | 250 (80.4) | 139 (81.3) | 111 (79.3) |  |  |  |  |
| Yes | 82 (24.3) | 30 (17.2) | 52 (31.7) | 71 (21.4) | 29 (16.9) | 42 (26.4) | 65 (19.9) | 29 (16.7) | 36 (23.5) | 61 (19.6) | 32 (18.7) | 29 (20.7) |  |  |  |  |
| **Had receptive anal sex with men** |  |  |  |  |  |  |  |  |  |  |  |  | 0.63 | 0.69 | 0.25 | 0.50 |
| No | 235 (69.5) | 128 (73.6) | 107 (65.2) | 237 (71.6) | 127 (73.8) | 110 (69.2) | 234 (71.6) | 121 (69.5) | 113 (73.9) | 230 (73.9) | 125 (73.1) | 105 (75.0) |  |  |  |  |
| Yes | 103 (30.5) | 46 (26.4) | 57 (34.8) | 94 (28.4) | 45 (26.2) | 49 (30.8) | 93 (28.4) | 53 (30.5) | 40 (26.1) | 81 (26.1) | 46 (26.9) | 35 (25.0) |  |  |  |  |
| **Had condomless anal sex with men** |  |  |  |  |  |  |  |  |  |  |  |  | 0.14 | 0.44 | 0.27 | 0.01 |
| No | 325 (96.2) | 171 (98.3) | 154 (93.9) | 323 (97.6) | 171 (99.4) | 152 (95.6) | 321 (98.2) | 173 (99.4) | 148 (96.7) | 307 (98.7) | 169 (98.8) | 138 (98.6) |  |  |  |  |
| Yes | 13 (3.8) | 3 (1.7) | 10 (6.1) | 8 (2.4) | 1 (0.6) | 7 (4.4) | 6 (1.8) | 1 (0.6) | 5 (3.3) | 4 (1.3) | 2 (1.2) | 2 (1.4) |  |  |  |  |
| **Had anal sex with known HIV-positive men** |  |  |  |  |  |  |  |  |  |  |  |  | 0.23 | 0.67 | 0.26 | 0.65 |
| No | 315 (93.2) | 163 (93.7) | 152 (92.7) | 304 (91.8) | 157 (91.3) | 147 (92.5) | 311 (95.1) | 164 (94.3) | 147 (96.1) | 296 (95.2) | 161 (94.2) | 135 (96.4) |  |  |  |  |
| Yes | 23 (6.8) | 11 (6.3) | 12 (7.3) | 27 (8.2) | 15 (8.7) | 12 (7.5) | 16 (4.9) | 10 (5.7) | 6 (3.9) | 15 (4.8) | 10 (5.8) | 5 (3.6) |  |  |  |  |
| **Had condomless vaginal sex with women** |  |  |  |  |  |  |  |  |  |  |  |  | 0.12 | 0.29 | 0.12 | 0.11 |
| No | 331 (97.9) | 171 (98.3) | 160 (97.6) | 320 (96.7) | 167 (97.1) | 153 (96.2) | 316 (96.6) | 172 (98.9) | 144 (94.1) | 304 (97.8) | 170 (99.4) | 134 (95.7) |  |  |  |  |
| Yes | 7 (2.1) | 3 (1.7) | 4 (2.4) | 11 (3.3) | 5 (2.9) | 6 (3.8) | 11 (3.4) | 2 (1.1) | 9 (5.9) | 7 (2.2) | 1 (0.6) | 6 (4.3) |  |  |  |  |

Note: sample size may vary due to missing response at each follow-up visit; SOC, standard-of-care

^a^ test for the overall trend, ^b^ test for the trend in intervention group, ^c^ test for the trend in control group, ^d^ comparing high-risk behaviors across intervention and control arms after accounting for repeated measures.
